# Management of type 2 diabetes in pregnancy: a narrative review

**DOI:** 10.3389/fendo.2023.1193271

**Published:** 2023-07-21

**Authors:** Lore Raets, Anne Ingelbrecht, Katrien Benhalima

**Affiliations:** ^1^ Department of Endocrinology, University Hospital Gasthuisberg, KU Leuven, Leuven, Belgium; ^2^ Department of Medicine, KU Leuven, Leuven, Belgium

**Keywords:** management, type 2 diabetes mellitus, pregnancy, preconception, peripartum, postpartum Frans (standard)

## Abstract

The prevalence of type 2 diabetes (T2DM) at reproductive age is rising. Women with T2DM have a similarly high risk for pregnancy complications as pregnant women with type 1 diabetes. To reduce adverse pregnancy and neonatal outcomes, such as preeclampsia and preterm delivery, a multi-target approach is necessary. Tight glycemic control together with appropriate gestational weight gain, lifestyle measures, and if necessary, antihypertensive treatment and low-dose aspirin is advised. This narrative review discusses the latest evidence on preconception care, management of diabetes-related complications, lifestyle counselling, recommendations on gestational weight gain, pharmacologic treatment and early postpartum management of T2DM.

## 1 Introduction

The prevalence of type 2 diabetes mellitus (T2DM) in pregnancy is increasing, mainly because of the rise in maternal obesity (1, 2). Pregestational diabetes occurs in one to two percent of all pregnancies (3). T2DM accounts for 30-50% of cases with pregestational diabetes in pregnancy (4, 5). Women with T2DM during pregnancy and their offspring are at increased risk for pregnancy complications (6). Risks for the mother include miscarriage, preeclampsia, gestational hypertension, maternal birth trauma, and caesarean delivery (7). Also, preterm delivery (<37 weeks) occurs four times more frequent compared to pregnancies of women without diabetes. Neonates of mothers with diabetes are more often large-for-gestational age (LGA), which is associated with birth trauma (shoulder dystocia), hypertrophic cardiomyopathy, neonatal respiratory problems, and metabolic complications (hypoglycaemia, hyperbilirubinemia, hypocalcaemia, and polycythaemia) (8). Despite the fact that women with T2DM have in general lower haemoglobin A1c (HbA1c) levels and a shorter diabetes duration compared to women with type 1 diabetes (T1DM), the risk for adverse pregnancy complications is similar compared to women with T1DM (9). This is in part related to the increased risk for co-morbidities in women with T2DM such as obesity, hypertension and the metabolic syndrome (9). In addition, pregnant women with youth-onset T2DM had very high rates of adverse pregnancy outcomes and higher rates of co-morbidities (10). T2DM is also associated with diabetes-related complications which can be classified as microvascular complications including nervous system damage (neuropathy), renal system damage (nephropathy) and eye damage (retinopathy) or macrovascular complications including cardiovascular disease (11). Contraindication for pregnancy are pre-existing low maternal renal function due to the increased risk for end-stage renal disease after pregnancy {Ringholm, 2016 #31}. Moreover, the risk for perinatal mortality seems to be higher in T2DM compared to T1DM (5, 12). Offspring of mothers with T2DM are also at increased risk for metabolic complications (such as T2DM and obesity) later in life (13).

**Figure 1:**
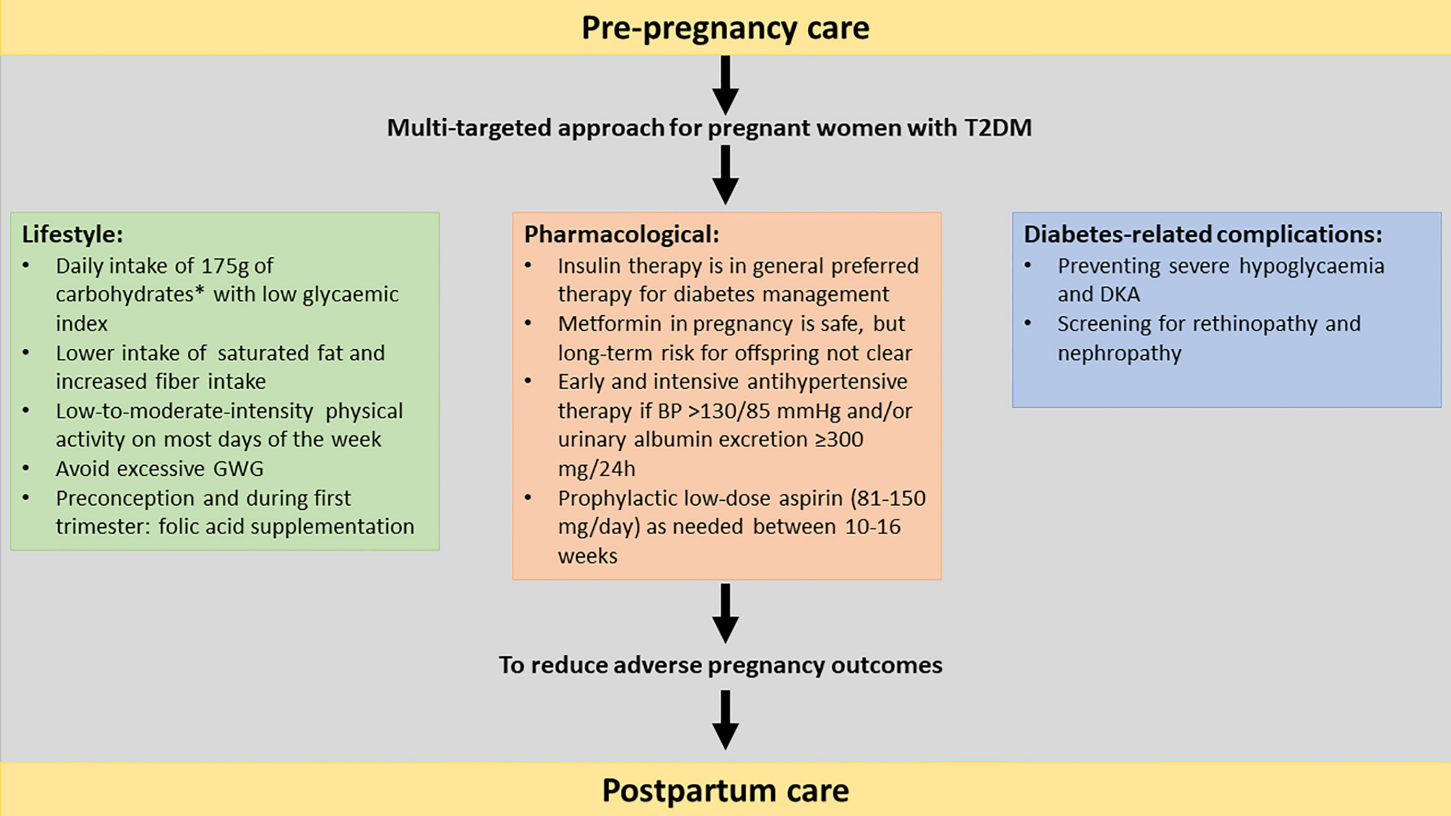
Multi-targeted approach of type 2 diabetes in pregnancy including pre-pregnancy care, lifestyle, pharmacological to reduce the risk for adverse pregnancy outcomes.T2DM, type 2 diabetes mellitus; GWG, gestational weight gain, OHA: oral hypoglycaemic agent; BP, blood pressure; DKA, diabetic ketoacidosis. *The recommended minimum intake of carbohydrates in pregnancy is uncertain due to limited evidence.

During pregnancy there is a state of increased insulin resistance as a result of a shift in hormones. In normal pregnancies beta-cell adaptation occurs, with an increasing beta-cell mass and function. In women with T2DM, the pre-existing insulin resistance is aggravated by pregnancy and since their beta- cell function is impaired, this might lead to more severe hyperglycaemia throughout pregnancy. During the second and third trimesters, a more pronounced increase in gluconeogenesis and lipolysis occurs in women with T2DM, leading to higher postprandial glucose spikes (9). Current diabetes management consists of a multitargeted approach to reduce the risk of adverse pregnancy outcomes. The most important management cornerstones are glycaemic control, dietary advice, limiting gestational weight gain (GWG), blood pressure control and easily accessible patient education (6). This narrative review provides an overview of the management of T2DM in pregnancy, including preconception care, management during pregnancy, peripartum and early postpartum (**Figure 1**). The use of new technologies (such as glucose sensors and insulin pumps) for the glycaemic management in pregnancy are not the scope of this review, as this has recently been reviewed in this journal (4).

## 2 Search strategy and selection criteria

**Figure 2:**
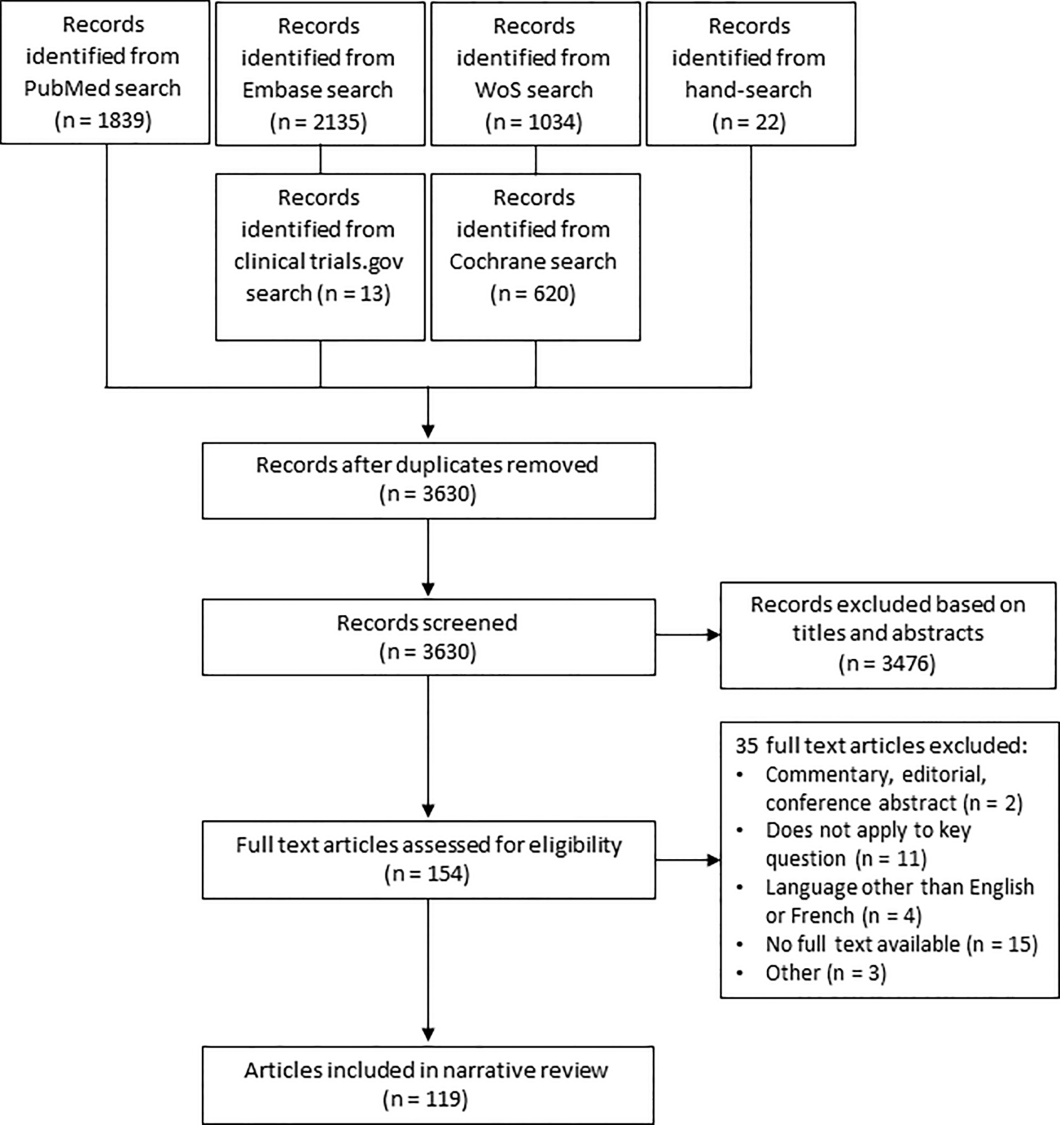
Flowchart literature search and selection process.

Between February 2022 and March 2023, a literature search was conducted on PubMed, Embase, Web Of Science and Cochrane library (Appendix I). Studies published from 2012 onwards were included. Cross-sectional studies, case-control studies, cohort studies, randomized controlled trials (RCT’s) and systematic reviews were considered for this narrative review. The study population were pregnant women with T2DM. The effects of the implementation of different protocols, guidelines or programs for management of T2DM were evaluated. Animal studies, descriptive designs (case series and case reports), and articles written in a language other than English, French or Dutch were excluded. As this is not a systematic review of the literature, we reported our results in a descriptive manner. We did not perform a meta-analysis. In addition to this, two reviewers (LR and AI) hand-searched the reference lists of the selected articles and reviews to identify further relevant articles. We identified 3630 articles of which 154 articles were selected as possibly relevant. 119 studies were included in this review after examination of the full text (**Figure 2**).

## 3 Results

### 3.1 Preconception management

When there is active pregnancy wish, women should receive preconception advice ideally once monthly (and at least every three months) (1). Preconception care should not only focus on glycaemic targets, but also on nutrition, diabetes education and screening for comorbidities and complications (14). Despite the beneficial effects of preconception care, women with T2DM are less likely to follow preconception programmes compared to women with T1DM (32% vs. 54% with T1DM), which is a missed opportunity to improve pregnancy outcomes (1, 4–6, 15).

#### 3.1.1 Glycemic control

Glycaemic control should be optimised before conception, as achieving tight glycaemic control in early pregnancy is crucial to reduce the risk for congenital anomalies, while maintaining strict glycaemic control throughout pregnancy is needed to reduce the risk for fetal overgrowth. The incidence of congenital malformations is linearly associated with increasing HbA1c values in early pregnancy (1). Pre-pregnancy care has demonstrated to lower HbA1c in the first trimester by an average of 1.92%. The downside was an increased risk of hypoglycaemia during the first trimester in women who received pre-pregnancy care (16). However, studies showed a risk reduction of congenital malformations by 71% and reduction of the perinatal mortality risk by 54% (17). In addition, pre-pregnancy care was associated with a reduction in preterm delivery rate of 15%, a risk reduction of 48% for small-for-gestational age (SGA) infants and a risk reduction of 25% for neonatal intensive care unit (NICU) admissions (17). The National Institute for Health and Care Excellence (NICE) and the American Diabetes Association (ADA) guidelines advise therefore to aim for HbA1c values <6.5% (<48 mmol/mol) preconception. Women with T2DM are therefore in generally advised not to get pregnant until the HbA1c value is <7.0% (<53 mmol/mol) because of the associated risks for congenital malformations, LGA, SGA, preterm deliveries and NICU admissions (14, 18).

#### 3.1.2 Medication review

Angiotensin-converting enzyme (ACE) inhibitors, angiotensin receptor blockers, and statins have possible teratogenic effects (19). Women who are on antihypertensive medication such as ACE inhibitors and angiotensin-II receptor antagonists should therefore be switched to suitable antihypertensive agents before conception, or as soon as pregnancy is confirmed (18). Antihypertensive agents that are considered save in pregnancy are methyldopa, labetalol, nifedipine or diltiazem (6). Insulin is the preferred agent for glycaemic control in T2DM women during pregnancy since it cannot cross the placenta. Metformin and glyburide are also safe to use during the first trimester (14). Advice on quitting alcohol, drugs and nicotine use should also be provided (1, 14).

There is no general consensus on the exact dose of folic acid to be administered during preconception in T2DM women. The NICE guideline suggests that women with diabetes should take 5mg of folic acid until 12 weeks of gestation (18). The ADA and the American College of Obstetricians and Gynaecologists (ACOG) guidelines both recommend a standard dose of folic acid of 400µg or 0.4mg prior to conception (3, 14) (**Table 1**). Women should be informed about the importance of folic acid intake, since women with T2DM show lower rates of use of preconception folic acid compared to women with T1DM (22.2% vs. 44.1%, p<0.0001) (5).

**Table 1:** recommendations of folic acid dosage and timing in diabetic pregnancy Data from the ACOG (3), ADA 2021 (14), ADIPS (20) and NICE (18) guidelines.

	Dosage	Timing
**ACOG** (3)	400 µg/day	Prior to conception
**ADA** (14)	400 µg/day	Prior to conception
**ADIPS** (20)	2.5-5 mg/day	Prior to conception to 12 weeks of gestation
**NICE** (18)	5 mg/day	Until 12 weeks of gestation

In addition to folic acid, pregnant women with diabetes should also take 1000 mg elemental calcium and 600 IU vitamin D daily (19). They should follow the same recommendations regarding minerals and vitamins as pregnant women without diabetes (21).

### 3.2 Management of diabetes-related complications

Diabetes is associated with microvascular complications such as diabetic retinopathy (DR) and nephropathy. The risk for progression of DR is higher in pregnancy compared to non-pregnancy populations (22). The main risk factors for development and progression of DR in pregnancy are poor glycaemic control, rapid lowering of glucose levels, longer diabetes duration and previous history of DR. Chronic hypertension, defined as pre-pregnancy hypertension or newly detected office blood pressure ≥135/85 mmHg with home blood pressure ≥130/80 mmHg in early pregnancy and pre-existing diabetic nephropathy are also associated with DR (23, 24). Studies have shown that the estimated prevalence of DR in early pregnancy in T2DM is around 14% (23, 25), while he prevalence of new onset DR was around 9.0% (26). The ADA suggests that the ophthalmological assessments should therefore take place before conception, during the first trimester, and further as needed during the second and third trimester of pregnancy, and within the first year postpartum as indicated by the degree of retinopathy (14, 27). The Australasian Diabetes in Pregnancy Society (ADIPS) guideline recommends retinal screening during the first trimester of pregnancy, unless already performed within 3 months prior to conception. The retinal assessment should be repeated at 28 weeks gestation if there is no DR perceived during the first retinal screening (20). A recent Danish study suggests that the frequency of retinal screening can be reduced in women with no DR in early pregnancy and good glycaemic control, defined as HbA1c <7% (<53mmol/mol). In this specific group, none of the women developed sight-threatening retinopathy and 94% remained without any degree of DR during pregnancy. In contrast, among women with mild DR in early pregnancy, development of sight-threatening retinopathy occurred in 8% (23).

Diabetic nephropathy is a progressive disease that affects approximately 30% of the non-pregnant diabetic population. Diabetic nephropathy is characterized by persistent proteinuria, hypertension, and a decline in glomerular filtration rate (GFR) (28). Nephropathy is defined as an urinary albumin-creatinine ratio ≥300 mg/g or microalbuminuria with an urinary albumin-creatinine ratio of 30–299 mg/g. During the course of pregnancy, albuminuria typically increases, and returns to or near pre-pregnancy baseline values after delivery (29). Pregnancy does generally not worsen the renal function in women with nephropathy but the degree of nephropathy can progress during pregnancy and is associated with higher rates of preeclampsia and preterm delivery (28–31). The ADA therefore recommends to screen for nephropathy in each trimester (14). Risk factors for the development of preeclampsia in this population are reduced kidney function, hypertension at the start of pregnancy, and nephrotic proteinuria (28, 30, 31). The cornerstones of nephropathy management during pregnancy are tight glycaemic management and antihypertensive treatment (14, 30–32). Contraindication for pregnancy in women with diabetic nephropathy is the presence of low kidney function, since pregnancy could lead to further deterioration leading to end-stage renal disease. A serum creatinine above 176 µmol/L is a predictor for the risk of pregnancy-induced decline in maternal kidney function. Therefore, women with pre-existing diabetes and nephropathy should always be counselled during preconception to determine the risk for both mother and foetus, to optimize glycaemic and nephrological status and to adjust medical treatment if necessary {Ringholm, 2016 #29}.

### 3.3 Lifestyle measurements

#### 3.3.1 Dietary advice

To optimize maternal nutrition, counseling by a dietician familiar with the management of T2DM in pregnancy should be suggested to women with T2DM who are pregnant. The amount and type of carbohydrate is particularly important in women with diabetes since simple carbohydrates will result in higher postprandial glycaemic excursions (14). To minimalize the risk for ketone formation, which might be associated with reduced offspring IQ on the long-term, a minimum daily intake of 175 g of carbohydrates is in general recommended in pregnancy (6, 14, 33). This is however based on limited evidence. For women with pregestational diabetes, the recommendation is 150 g from the main carbohydrate sources (such as bread, pasta, dairy products and fruits) and 25g from other carbohydrate source (vegetables) (6, 33). Recommended carbohydrate sources include fresh vegetables, some fruits and whole grains (1). Obese women with diabetes are also recommended to reduce their calorie intake by approximately one-third (compared with their usual intake before pregnancy) while maintaining a minimum intake of 1600 to 1800 kcal. Furthermore, carbohydrate intake should be limited to 35% to 45% of total calories (21). In addition, studies have shown that elevated triglycerides and low HDL-cholesterol in first and third trimester are predictors for LGA infants (34–36). A combination of weight management before pregnancy, reducing gestational weight gain, reducing maternal triglyceride levels by a low-fat diet with minimizing intake of saturated fat and a higher fiber intake may result in fewer cases of excessive fetal growth and lower percentage of preeclampsia (34, 36, 37). However, there are to date no RCT’s exploring the management of maternal lipids in pregnant women with T2DM.

#### 3.3.2 Physical activity

There is strong evidence that exercise improves insulin resistance and can lower HbA1c values in the general population with T2DM. However, the specific benefits of physical activity for pregnant women with T2DM are less clear due to lack of evidence from large RCT’s. In a prospective cohort study, women with diabetes who showed more sedentary behaviour early in pregnancy had a higher risk of developing preeclampsia later in pregnancy, even though the total physical activity was similar. However, sedentary behaviour was no longer an independent predictor of preeclampsia in the multiple regression analysis, highlighting the need for more evidence. Studies in women with gestational diabetes (GDM) suggest that low-to-moderate-intensity activities improves blood glucose and is safe during pregnancy (38). The ADIPS guideline suggests therefore that women with pre-existing diabetes should perform approximately 30 min of low-to-moderate-intensity physical activity on most days of the week, in line with the recommendations for a general non-pregnant population (20).

#### 3.3.3 Weight management before pregnancy

T2DM is often associated with obesity (14). The prevalence of T2DM increases in parallel with the prevalence of obesity (4, 5, 39). Maternal obesity in women with pre-existing diabetes has been shown to be an independent risk factor for adverse pregnancy outcomes such as caesarean section, NICU admission, LGA infants, and preterm birth (40). In addition, obesity is an independent risk factor for congenital heart defects and central nervous system malformation (41, 42). Pregnancy management of obese women with T2DM should therefore start before conception, including weight loss counselling and screening for obstructive sleep apnoea (OSA). A 10% decrease in pre-pregnancy BMI has been associated with at least a 10% lower risk of preeclampsia, medically indicated preterm birth, macrosomia, and stillbirth (39). The importance of prepregnancy weight management in obese women was also demonstrated in women without diabetes, with higher rates of preeclampsia and LGA in women with preconception and between pregnancies weight gain (43).

In addition, more women of reproductive age have severe obesity (BMI≥ 35kg/m^2^). In these women, bariatric surgery (BS) could be beneficial, with often an increase in fertility (44–46). However, pregnancy after BS holds both benefits as well as possible harms for mother and child. Women who underwent BS had a lower risk of LGA infants and hypertensive disorders, but a higher risk for SGA infants (44, 46, 47). To prevent the risk for SGA infants, women should be informed that pregnancy shortly after BS is not recommended and should be delayed until at least 12-18 months after BS or until stabilization of weight occurs (45, 48). Therefore, it is important that these women will receive information and follow-up during preconception. In addition, a close follow-up of micronutrients and glucose during pregnancy is recommended to avoid adverse pregnancy outcomes such as SGA infants, growth restriction and preterm delivery (44, 46).

##### 3.3.3.1 Gestational weight gain

A recent study showed that in women with pregestational and GDM, pregnancies with excessive gestational weight gain (GWG) were at increased risk for caesarean delivery, preeclampsia, LGA, and macrosomia, compared with those within the ‘National Academy of Medicine’ (NAM, also known as Institute of Medicine, IOM) recommendations (6, 49) (**Table 2**). Restricted GWG (≤5 kg) in obese (BMI ≥ 30) women with T2DM was associated with lower rates of LGA and similar rates of SGA compared to women with GWG >5 kg. Furthermore, there was a reduced risk of perinatal morbidity and more often delivery closer to term in this group (6, 50). In obese women, no association was found between restricted GWG and SGA infants (51). In general, a GWG within or at the lower end of the scale of the NAM 2009 guidelines is recommended to reduce the risk for LGA infants (6, 27, 52–55). More strict GWG guidelines have been proposed in Denmark (54). Structured weight management programs and dietary advice during pregnancy are needed to avoid excessive GWG (6, 12, 54, 56).

**Table 2:** The National Academy of Medicine (2009) and Copenhagen Guidelines for total gestational weight gain.

BMI preconceptionally	The National Academy of Medicine guidelines for total gestational weight gain	The Copenhagen Guidelines for total gestational weight gain
Underweight women (BMI <18.5kg/m²)	12.5-18.0 kg	No information
Normal weight women (BMI 18.5-24.9kg/m²)	11.5-16.0 kg	10.0-15.0 kg
Overweight women (BMI 25.0-29.9kg/m²)	7.0-11.5 kg	5.0-8.0 kg
Obese women (BMI ≥30kg/m²)	5.0-9.0 kg	0-5.0 kg

#### 3.3.4 Group education and telemedicine

The benefits of group education are multiple: improved knowledge acquisition, behaviour modification *via* social normalization, enhanced social support, increased time spent with a provider, and shared knowledge among a peer group. A recent RCT in women with T2DM or GDM, explored the effect of group prenatal care on the completion of diabetes self-care activities, including diet, exercise, blood sugar testing, and medication adherence. In comparison to individual education, participants receiving group education had less GWG and better adherence to dietary advices (57). Patient satisfaction also appeared to be higher in women enrolled in group care (58).

Telemedicine could be an aid in the management of diabetes in pregnancy, to stimulate self-management in between the visits at the out-patient clinic. However, a recent meta-analysis showed no improvement of maternal and fetal outcomes when using telemedicine compared to usual care. Further research is needed, including economic evaluations (59). A good example of a helpful app is ‘Pregnant with Diabetes’, developed by Danish researchers which gives evidence-based clinical recommendations and was launched in 2014 (60). This app is available free of charge in Danish, English and Swedish. This app can help to provide support and suitable information for women with diabetes when planning pregnancy, but also during pregnancy. This app can also be combined with other apps, such as carbohydrate counting apps and apps calculating meal-time insulin dose to individualize the carbohydrate-to-insulin ration (6).

### 3.4 Glycaemic targets during pregnancy

HbA1c levels should be maintained below 6.0% (42 mmol/mol) in pregnancy, if this level can be achieved without significant maternal hypoglycaemia. Higher HbA1c levels are associated with an increased risk of congenital malformations, LGA, preeclampsia and preterm delivery (6). Glucose should be measured *via* capillary blood monitoring at least fasting, 1-hour and 2-hour postprandial. Recommended glucose levels are fasting 70-95 mg/dL (3.9-5.3 mmol/L) and either 1-hour postprandial glucose levels between 110-140 mg/dL (6.1-7.8 mmol/L) or 2-hour postprandial glucose levels 100-120 mg/dL (5.6-6.7 mmol/L) (3, 14, 18, 20) (**Table 3**). Postprandial glucose values are associated with foetal growth and thus with the risk of macrosomia. An average postprandial glucose value of 120mg/dl was associated with a 20% risk of getting a macrosomic neonate (61).

**Table 3:** recommended glucose and HbA1c targets during pregnancy * if this can be achieved without significant hypoglycaemia Data from the ACOG (3), ADA 2022 (14), ADIPS (20) and NICE (18) guidelines.

	Recommended glucose targets	HbA1c
**ACOG** (3)	Fasting and premeal glucose 95 mg/dL (5.3 mmol/L) or less and either 1-hour postprandial glucose 140 mg/dL (7.8 mmol/L) or less or 2-hour postprandial values of 120 mg/dL (6.7 mmol/L) or less. During the night, glucose levels should not decrease to less than 60 mg/dL (3.3 mmol/L)	<6.0% (42 mmol/mol)*
**ADA** (14)	Fasting glucose 70–95 mg/dL (3.9–5.3 mmol/L) and either 1-hour postprandial glucose 110– 140 mg/dL (6.1–7.8 mmol/L) or 2-hour postprandial glucose 100– 120 mg/dL (5.6–6.7 mmol/L)	<6.0% (42 mmol/mol)*
**ADIPS** (20)	Fasting and preprandial 72-95 mg/dL (4.0-5.3 mmol/L) 1-hour postprandial 99-140 mg/dL (5.5-7.8 mmol/L) 2-hour postprandial 90-120 mg/dL (5.0-6.7mmol/L)	≤6.5% (48 mmol/mol)* preconception and during 1st trimester ≤6.0% (42 mmol/mol)* during 2nd and 3rd trimester
**NICE** (18)	Fasting 95 mg/dL (5.3 mmol/L) and 1-hour postprandial 140 mg/dL (7.8 mmol/L) or 2-hours postprandial 115 mg/dL (6.4 mmol/L)	≤6.5% (48 mmol/mol)*

Severe maternal hypoglycaemia (<54 mg/dl or <3.0 mmol/L) occurred in 19% of the pregnant women with T2DM who were treated with basal-bolus or pre-mixed insulin from early pregnancy to delivery. As during pregnancy a strict glycaemic control is aimed for, this can be associated with an increased risk for severe hypoglycaemia (6, 62). Risk factors for severe hypoglycaemia during pregnancy are impaired hypoglycaemia awareness, and long duration of diabetes. In addition, the risk for repeated episodes of severe hypoglycaemia was positively correlated to the presence of peripheral neuropathy (63).

Although diabetic ketoacidosis (DKA) occurs more frequently in pregnant women with T1DM, it can also occur in women with T2DM. Risk factors for DKA are an infection, vomiting, and use of beta-mimetic agents (64). When glycaemia exceeds 200 mg/dL, blood ketone levels need to be determined (3). Blood ketones >0.6 warrant medical advice (20). Attention must be paid to the occurrence of DKA at lower blood glucose levels than in non-pregnant women, since there is a higher glomerular filtration and lower renal threshold of glycosuria during pregnancy (65). Thus, DKA can occur in absence of hyperglycaemia and should always be ruled out in pregnant women with diabetes with persistent nausea and vomiting (64). The management of DKA consists of fluid resuscitation, electrolyte replacement and intravenous insulin (65).

### 3.5 Pharmacological management

#### 3.5.1 Hypertension management

Women with diabetes are at increased risk for uncontrolled maternal hypertension and consequently impaired foetal growth (14). When blood pressure (BP) exceeds 130/80 mmHg prior to or during the first trimester, chronic hypertension should be suspected. Preeclampsia is characterized by hypertension (BP ≥140/90 mmHg after 20 weeks), proteinuria, and peripheral oedema (28). The estimated rate of preeclampsia in women with diabetes is 20% (66, 67). A prospective cohort study showed that diastolic BP and the presence of diabetic microangiopathy at the first antenatal visit were independent risk factors for developing preeclampsia later in pregnancy (7). The ADA recommends a target goal BP of 110–135/85 mmHg in pregnancies complicated by diabetes and chronic hypertension (14, 20). The 2019 NICE and ADIPS guidelines recommend a target BP of 135/85 mmHg for pregnant women with chronic hypertension, gestational hypertension or preeclampsia (68).

##### 3.5.1.1 Low-dose aspirin

Women with diabetes during pregnancy could have an increased risk for developing preeclampsia. For pregnant women with this increased risk, aspirin is often prescribed (69). The ASPRE study, which is the largest RCT up to now in women with increased risk for preeclampsia, demonstrated that treatment with aspirin at 150 mg per day reduced the risk for preterm preeclampsia (<37 weeks) with an odds ratio of 0.38 compared to placebo. Nonetheless, very few women in the study had pregestational diabetes (70). The use of aspirin in pregnant women with diabetes has only been studied in two smaller RCT’s, but they could not find a reduction for preeclampsia (71, 72). Moreover, these studies evaluated the treatment with aspirin mostly initiated during the second trimester. This suggests that aspirin therapy may be more favourable if started during the first trimester. A Danish prospective cohort study demonstrated no risk reduction in pregnant women with preexisting diabetes if prophylactic aspirin therapy was implemented in all women compared to the risk-based prophylaxis strategy, However, this study was not randomized (69). The ongoing multicentre double-blinded placebo-controlled RCT from Ireland (IRELAND study) was specifically designed to determine the potential beneficial effect of aspirin initiated in the first trimester until 36 weeks of gestation in women with pre-existing diabetes. The primary outcomes of this study will be placental dysfunction (preeclampsia, preterm birth before 34 weeks, birthweight below the 10th centile or perinatal mortality) (66).

Although there is a paucity of data on the use of aspirin in pregnancies with diabetes, the ACOG, ADA, ADIPS and NICE guidelines (**Table 4**) all recommend to initiate aspirin prophylaxis since pre-existing diabetes is considered a high-risk factor for the development of preeclampsia. In general, a dose of 100-150 mg/dL is recommended, starting from 12-16 weeks of gestation up to 36 weeks (3, 14, 20, 68, 70). Low-dose aspirin treatment should certainly be advised in women with diabetes nephropathy or microalbuminuria in order to prevent cardiovascular events and to reduce the risk of preeclampsia (31).

**Table 4:** recommendations of aspirin dosage and timing in diabetic pregnancy Data from the ACOG (3), ADA 2022 (14), ADIPS (20) and NICE (68) guidelines.

	Dosage	Timing
**ACOG** (3)	81 mg/day	Start at 12-28 weeks and continue until delivery
**ADA** (14)	100-150 mg/day	Start at 12-16 weeks of gestation
**ADIPS** (20)	100-150 mg/day	From 12 weeks to 36 weeks of gestation
**NICE** (68)	75-150 mg/day	From 12 weeks until delivery

#### 3.5.2 Diabetes medication

##### 3.5.2.1 Insulin

Intensive insulin therapy is the first choice therapy for achieving strict glycaemic control during pregnancy (6, 14, 73). Short-acting insulin analogues are favoured over human insulins as this leads to more flexibility and lower the risk for hypoglycaemia (74). In addition, long-acting insulin analogues are used to lower the risk for (nocturnal) hypoglycaemia and have a longer-acting mode of action (compared to human insulin) of 24-42 hours (75).

The short-acting insulin analogues insulin lispro and aspart were found to have acceptable safety profiles, minimal transplacental transfer, and no evidence of teratogenicity (6, 76, 77). A meta-analysis found insulin aspart, glargine, and detemir to be safe during pregnancy, with no increase in maternal or foetal complications. Insulin lispro, however, was associated with an increase in LGA infants (78). There is an European approval for the use of ultra-rapid acting aspart (Fiasp^®^) and rapid-acting lispro (Lyumjev^®^) in pregnancy. The only difference between both is the inclusion of ‘Generally regarded as safe’ ingredients (79, 80). Currently, there is an ongoing RCT, the CopenFast trial, comparing Fiasp^®^ to insulin NovoRapid^®^ in the treatment of women with T1DM or T2DM during pregnancy and lactation (81). Results are expected for later in 2023. Glulisine is not allowed for use in pregnancy, because of the lack of large studies (6, 75). In early pregnancy, short-acting insulin analogues should be injected at least 15 minutes before eating. By the time of late pregnancy, this should be extended to 30-45 minutes before meals to account for increased post-meal insulin resistance and delayed insulin absorption with advancing gestation (82){Benhalima, 2023 #164}.

A large RCT demonstrated that the long-acting insulin analogue detemir leads to lower fasting glycaemia, with similar HbA1_c_ and similar rates of hypoglycaemia compared to neutral protamine Hagedorn insulin (83). RCT’s on the use of glargine (Lantus^®^) in pregnancy are lacking, but observational data have shown that it is safe to use both glargine U100 and glargine U300 during pregnancy (75, 84). Very recently, the EXPECT trial, a large multicentre RCT comparing degludec with detemir (both in combination with insulin aspart) in pregnant women with T1DM, showed that degludec was non-inferior to detemir with similar glycaemic control and pregnancy outcomes (85). Based on this RCT, degludec has recently been approved for use in pregnancy in Europe, the USA and Canada.

##### 3.5.2.2 Oral hypoglycaemic agents

Oral hypoglycaemic agents (OHA) have the advantage that no injections are needed and can therefore be a more attractive option for pregnant women with T2DM (86). However, women who are treated with diabetes medication such as dipeptidyl peptidase 4 inhibitors, sodium-glucose cotransporter 2 inhibitors, meglitinides, thiazolidinediones and injectable glucagon-like peptide 1 agonists, are recommended to stop this treatment and switch to insulin prior to conception (6, 14, 20). These OHA’s are not approved for use in pregnancy as more evidence is needed regarding the safety of these non-insulin antidiabetics during pregnancy (87–91).

Metformin and glyburide (sulfonylurea) are safe to use during pregnancy, because they are not related to teratogenic effects (92). However, meta-analyses have indicated that pregnant women who continue to use sulfonylurea have a higher risk for neonates with macrosomia and neonatal hypoglycaemia compared to pregnant women who use insulin (92, 93).

Women with T2DM are often treated with metformin before pregnancy and this also often continued during pregnancy. The MiTy trial was a large multicentric RCT comparing the addition of metformin to insulin with placebo in pregnant women with T2DM and showed that the addition of metformin might have several advantages in T2DM pregnancy, with fewer episodes of hypoglycaemia, lower total insulin dose and fewer caesarean births, lower risk for LGA infants and less neonatal (2). The ongoing MOMPOD RCT, randomizing pregnant women with T2DM to insulin plus metformin or placebo, will also explore the effect of additional metformin therapy on composite adverse neonatal outcomes (94). In addition, several studies have demonstrated that treatment with metformin might be associated with less gestational weight gain compared to insulin (2, 95–98). These beneficial results were also shown in women with GDM and women with polycystic ovarian syndrome (PCOS) (99–101). However, there were more SGA babies in the metformin treated group (97, 102). The higher rates of SGA infants could be due to metformin exposure because metformin crosses the placenta, or could be mediated through secondary reasons such as improvements in glycaemic control and less GWG (103). SGA neonates were mainly seen in pregnant women with T2DM with nephropathy or hypertension. Therefore, treatment with metformin in this subgroup is not recommended (104). The EMA recently approved use of metformin during pregnancy. However, the long-term risk for the offspring exposed to metformin during pregnancy remains unclear. Several studies suggest that exposure to metformin *in utero* might be associated with an increased risk for a long-term adverse metabolic profile in the offspring with a higher BMI (105). In addition, the effect of epigenetic modifications on gene expression due to metformin are unclear, which requires further investigation. Study in mice showed that the epigenetic effects of metformin could result in adverse metabolic outcomes in offspring (such as impaired hepatic function and higher BMI) (106). Very recently, follow-up data of the MiTy trial showed no differences in anthropometrics between children exposed to metformin *in utero* and those not exposed over a period of 24 months postpartum (107, 108). However, longer-term studies are needed as an increased BMI in the offspring exposed to metformin was generally seen in children ≥10 years. The MiTy trial is therefore also planning a longer follow-up study 5-10 years postpartum to investigate whether these children have an increased risk for obesity and T2DM.

#### 3.5.3 Glycemic management after antenatal corticosteroid therapy

Antenatal corticosteroid treatment (ACS) is used to promote foetal lung maturation in case of threatened preterm delivery. According to the NICE guidelines, diabetes should not be considered a contraindication to ACS (18). The conventional antenatal corticosteroid regimes betamethasone 12 mg IM q24h x 2 doses, or dexamethasone 6 mg IM q12h x 4 doses, can also be used in women with diabetes. However, as ACS has an important impact on glycaemic control, glycaemia should be strictly monitored with a 7 point profile (3 pre- meal, 3 post-meal estimations, and a 3 am value). The glycaemic management after ACS requires an individualized approach. In patients with T2DM an increased insulin requirement of 26-64% has been reported (109). The 2021 ‘Joint British Diabetes Society for Inpatient Care’ guidelines proposes two options, either to increase the basal and prandial insulin doses by 50% to 80%, or a variable rate intravenous insulin infusion (VRIII) should be started. VRIII should only be considered if blood glucose targets are outside the target range on two consecutive occasions in spite of increased insulin doses (typically by ~50%). The glucose target can be either the NICE target (72-140 mg/dL or 4.0–7.8 mmol/L) or the more liberal target range (90-144 mg/dL or 5.0–8.0 mmol/L) to limit the risk for maternal hypoglycaemia (110).

#### 3.5.4 Peripartum glycemic control

A peripartum diabetes management plan, including the blood glucose target zone, should be documented and agreed on for all pregnant women with diabetes (110). Since tight glucose targets intrapartum can also be associated with disadvantages (such as maternal hypoglycaemia), the ‘Joint British Diabetes Society for Inpatient Care’ suggests two types of intrapartum glycaemic targets: either tight 72-126 mg/dL (4.0-7.0 mmol/L) or more pragmatic glucose targets of 90-144 mg/dL (5.0-8.0 mmol/L). No significant association between in-target intrapartum glucose control and neonatal hypoglycaemia (regardless of diabetes type) was found (111, 112). In an operative delivery setting (caesarean section), a glycaemic target range of 90-144 mg/dL (5.0-8.0 mmol/L) is suggested. If intrapartum glucose levels are higher than 144 mg/dL on two consecutive occasions, a variable rate of VRIII is recommended. Alongside with the VRIII, an intravenous drip with 0.9% NaCl with 5% glucose and 0.15% KCl (20 mmol/L) or 0.3% KCl (40 mmol/L) at 50 ml/hr should be given (110). The sodium-rich solution reduces the risk of developing hyponatraemia (18, 113).

Because of a drop of insulin resistance immediately after delivery, a reduction of insulin dosage byat least 50% is recommended. In women who were not on insulin treatment before pregnancy, insulin infusion should be stopped after delivery (18, 110). Insulin can often be stopped after delivery in women with T2DM, but medical treatment with OAH remains necessary (114).

### 3.6 Monitoring fetal growth and wellbeing

The 2020 NICE guidelines suggest ultrasound monitoring of foetal growth and amniotic fluid volume every 4 weeks from 28 to 36 weeks in women with diabetes. Monitoring of foetal wellbeing (including methods such as foetal umbilical artery doppler recording, foetal heart rate recording and biophysical profile testing) is in generally not recommended before 38 weeks, and should only be done when foetal growth restriction is suspected. Women with diabetes who are at risk of foetal growth restriction, because of nephropathy of macrovascular disease, should be provided an individualised approach to monitoring of foetal growth (18). As the risk for stillbirth and SGA is six-times higher in women with pregestational diabetes, and two-fold increased for LGA babies (115), adequate monitoring of foetal growth is important in women with T2DM. However, estimating foetal weight by ultrasound is more difficult in pregnancies with diabetes since the fat in the foetus is often more disproportionally distributed. This can lead to a measurement error up to 900 grams on estimated foetal weight. Abdominal circumference is one of the most accurate parameters to assess foetal weight. When the abdominal circumference is greater than 90^th^ centile, macrosomia is present in up to 80% of neonates (116). In pregnancies without diabetes doppler flow measurements in foetal vessels (such as the umbilical artery) can be used to identify placental insufficiency. However, in pregnancies with diabetes, doppler studies are neither appropriate nor sufficient because of soft tissue overgrowth, major metabolic changes and a different vascular diameter. Instead, ultrasound imaging of the ductus venosus and foetal hepatic artery could play a role in identifying adverse perinatal outcomes in pregnancies with diabetes (116).

### 3.7 Timing of delivery

At present, there is no consensus on the optimal timing for delivering women with diabetes. In women with GDM, delivery between 38-40 weeks of gestation is generally safe. However, since women with T1DM and T2DM are at higher risk for stillbirth, delivery is often not delayed beyond 38 weeks (117). However, in women with T2DM without medical complications and/or good glycaemic control, lose antenatal monitoring and a scheduled delivery at 39 weeks gestation is suggested.

The rate of emergency caesarean section in pregnancies complicated by diabetes is three to four times higher compared to women without diabetes. Predictors of an emergency caesarean section, are nulliparity, presence of a hypertensive disorder, shorter maternal height and previous caesarean section. No association was found between maternal HbA1c, ultrasonically estimated foetal size in late pregnancy and risk for caesarean section in women with pre-existing diabetes (118).

### 3.8 Postpartum management

#### 3.8.1 Breastfeeding

Breastfeeding should be encouraged in women with T2DM because of multiple benefits such as lower rates of obesity, facilitation of postpartum weight loss. and a lower risk in the offspring to develop diabetes later in life (1). However, women with diabetes are less likely to initiate breastfeeding, so good education on benefits of breastfeeding is necessary (27). In addition, as breastfeeding reduces insulin requirements by 10 to 20%, insulin needs to be further reduced postpartum in women who exclusively breastfeed and additional carbohydrate snacks might be needed (40). The Endocrine Society and NICE suggest that metformin or glyburide therapy can be continued while breastfeeding if necessary (18, 21). However, caution is needed as low doses of metformin have been demonstrated in the milk (about 0.6% of the dose taken by mothers) and neonates are therefore exposed to metformin while breastfeeding. Antihypertensive medications such ACE inhibitors (captopril, enalapril and quinapril), calcium channel antagonists and beta-blockers (such as labetalol and propranolol) are considered safe during breastfeeding. Because of insufficient data during lactation, the use of angiotensin receptor blockers is not recommended (31).

#### 3.8.2 Contraception counselling

Contraception should already be discussed during pregnancy, such as the option of tubal ligation during caesarean section. Pre-existing diabetes is not a contraindication to any method of contraception (20). However, since T2DM is associated with obesity, insulin resistance and cardiovascular risk factors, contraception must be prescribed with caution. Combined oral contraceptives are known to increase the risk of stroke and myocardial infarction in women with diabetes. In addition, combined oral contraceptives can increase triglycerides in women with diabetes. Combined oral contraceptives should therefore preferably only be prescribed to women with a BMI <30 kg/m², without additional cardiovascular risk factors, or microvascular and/or cardiovascular complications. The use of progestin-only pills or non-hormonal contraceptives are than the preferred option (119).

## 4 Conclusion

The number of pregnancies in women with T2DM are increasing. Women with T2DM remain at increased risk for adverse pregnancy complications. However, these women are less likely to receive preconception management and planning. A multitargeted approach is needed with strict glycemic management, lifestyle counselling, screening for diabetes complications, hypertension management and low-dose aspirin as needed. Breastfeeding should be encouraged as this is associated with multiple benefits, such as less postpartum weight retention. We recommend a structured follow-up of women with T2DM in pregnancy, starting at preconception. In our center in Belgium, women with pregnancy wish are seen once per month when planning pregnancy, to obtain a HbA1c < 7.0% before conception and to provide information on nutrition and weight management before pregnancy, as well as proper diabetes education. Once these women are pregnant, follow-up is intensified to at least every two weeks. A multidisciplinary approach is needed with strict glycemic control as well as proper diabetes education, dietary follow-up, weight management, hypertension management and low-dose aspirin as needed. This multi-target approach requires close collaboration between endocrinologists, nurses, dietitians, obstetricians and midwives specialized in the management ofdiabetes in pregnancy. In addition, psychosocial support should be offered as needed.

In the future, more research is needed to review the effects of newer insulin analogues on glycemic control and adverse pregnancy outcomes. In addition, larger studies are needed to improve antihypertensive treatment during pregnancy. Also, well-designed studies investigating the usefulness of smartphone applications and telemedicine for diabetes education, adherence to therapy and motivation for adherence to lifestyle changes are needed.

## Author contributions

LR, AI and KB wrote the manuscript. All authors have read and agreed to the published version of the manuscript.
